# Medication Abortion Safety and Effectiveness With Misoprostol Alone

**DOI:** 10.1001/jamanetworkopen.2023.40042

**Published:** 2023-10-27

**Authors:** Ruvani Jayaweera, Ijeoma Egwuatu, Sybil Nmezi, Ika Ayu Kristianingrum, Ruth Zurbriggen, Belén Grosso, Chiara Bercu, Caitlin Gerdts, Heidi Moseson

**Affiliations:** 1 Ibis Reproductive Health, Oakland, California; 2 Generation Initiative Women and Youth Network, Lagos, Nigeria; 3 La Revuelta Colectiva Feminista, Neuquén, Argentina

## Abstract

**Importance:**

Misoprostol-alone regimens for abortion may be more effective than previously thought.

**Objective:**

To estimate the effectiveness of medication abortion with misoprostol alone among individuals self-managing their abortion.

**Design, Setting, and Participants:**

For this prospective observational cohort study of callers to safe abortion hotlines and accompaniment groups in Argentina, Nigeria, and Southeast Asia, participants were recruited between July 31, 2019, and October 1, 2020, prior to starting their medication abortion. Eligible participants were 13 years or older, had no contraindications to medication abortion, and were not currently bleeding. Participants completed a baseline and 2 follow-up surveys. The analysis was restricted to participants who reported using misoprostol alone and was performed between January 6, 2022 and September 8, 2023.

**Exposure:**

Self-managed medication abortion using misoprostol alone.

**Main Outcomes and Measures:**

The primary outcome was effectiveness, defined as participant self-report of complete abortion without procedural intervention, measured at 1 week and 3 weeks after taking misoprostol. Secondary outcomes included method safety, measured by self-report of experiencing warning signs (eg, heavy bleeding, pain, fever, discharge) indicative of a potential complication and by medical treatment (eg, blood transfusion, intravenous fluids, overnight hospital stay) indicative of a potential adverse event. Additional outcomes included length of bleeding and cramping, time to expulsion, and experience of adverse effects.

**Results:**

Among 1352 enrolled participants, 637 used misoprostol-alone regimens for abortion and were included in the analysis (591 [92.8%] from Nigeria, 45 [7.1%] from Southeast Asia, and 1 [0.2%] from Argentina; 384 [60.2%] aged 20-29 years; 317 [49.8%] with pregnancy durations <7 weeks and 205 [32.2%] with pregnancy durations between 7 and <9 weeks). At last follow-up after taking medication (median, 22 days; IQR, 21-26 days), 625 participants (98.1%; 95% CI, 96.7%-98.9%) had a complete abortion without procedural intervention. Potential adverse events were reported by 6 participants (0.9%; 95% CI, 0.4%-2.1%). Most participants experienced bleeding for less than 1 week (median, 4 days; IQR, 3-6 days) and expelled their pregnancy within 24 hours of starting the abortion process (median, 12 hours; IQR, 9-15 hours). Common side effects included nausea (335 participants [52.6%]), fever (232 [36.4%]), and diarrhea (181 [28.4%]).

**Conclusions and Relevance:**

The findings suggest that misoprostol alone is a highly effective method of pregnancy termination. Future research should explore strategies to maximize the effectiveness of misoprostol alone in clinical and nonclinical settings.

## Key Points

**Question:**

What is the effectiveness of self-managed medication abortion using misoprostol alone?

**Findings:**

In this cohort study of 637 callers to safe abortion hotlines and accompaniment groups, almost all (98.1%) had a complete abortion using misoprostol without procedural intervention.

**Meaning:**

These findings suggest that the effectiveness of misoprostol alone for abortion may be higher than previously thought, and exploration of expanding access via this regimen is warranted in both clinical and nonclinical contexts.

## Teaser

This cohort study examines the safety and effectiveness of misoprostol alone for self-managed abortion as reported by callers to safe abortion hotlines.

## Introduction

There are 2 medication regimens recommended by the World Health Organization for abortion: mifepristone in combination with misoprostol and misoprostol alone.^1^ Both regimens are highly effective, with low rates of complications.^2,3^ Clinical evidence has suggested that misoprostol alone is less effective than the combined regimen (approximately 80% vs approximately 95%),^2,3^ and many clinical guidelines recommend misoprostol alone only if mifepristone is unavailable.^4,5^ As a result, the combined regimen has long been the preferred method for medication abortion in countries where mifepristone is registered as a pharmaceutical product.

In settings where mifepristone is inaccessible, most medication abortions are performed using misoprostol alone.^6^ Misoprostol has many advantages, including low cost, a wide range of indications, availability in many settings without a prescription, ease of administration, and shelf stability.^7^ As a result, misoprostol for self-managed abortion (defined as when a person does something to end their own pregnancy without clinical supervision) has risen globally.^8^ Many models of care exist for providing information and support to people who cannot or do not want to access clinical care, such as community-based distribution,^9,10^ feminist accompaniment and safe abortion hotline models,^11,12^ and online telemedicine services.^13^

The aim of much of the clinical literature on misoprostol alone was to establish recommended dosing and administration routes; as a result, clinical data on the effectiveness of currently endorsed misoprostol-alone protocols are sparse.^2,14^ There are only 2 clinical trials on misoprostol-alone regimens that use the currently endorsed regimen: 800-μg misoprostol tablets administered sublingually, vaginally, or buccally every 3 hours for at least 3 doses (2400 μg total).^4,15^ In a 2007 study, 84% of 1025 participants with pregnancy duration less than 9 weeks randomized to an arm following this regimen had a complete abortion without procedural intervention at 2-week follow-up.^16^ In a 2019 study, 93% of 388 participants with pregnancy duration less than 10 weeks randomized to an arm following this regimen had a complete abortion at 1-week follow-up.^17^ While there are a growing number of prospective studies of self-managed misoprostol-alone use,^9,10,18,19,20^ only 2 documented the currently endorsed misoprostol-alone regimen. In a pilot study from Nigeria and Argentina of 94 participants with pregnancy durations less than 17 weeks (86% had pregnancy durations <10 weeks), 94% had a complete abortion at 3 week follow-up.^19^ A study of 568 participants in the US with pregnancy durations less than 10 weeks found effectiveness to be 87% at 4 week follow-up.^20^ Additional data with a shorter evaluation time and information on physical experience and health care seeking are needed from self-managed contexts.

Additional research is needed on the currently endorsed misoprostol-alone regimen, particularly given the lack of detailed information about the physical experience of using misoprostol alone.^6^ The aim of this study was to estimate the safety and effectiveness of misoprostol alone for abortion from a sample of callers to safe abortion hotlines and accompaniment groups and to document participants’ physical experiences and health care–seeking experiences. Findings from this study may inform counseling and support for out-of-clinic models, as well as clinical policy and practice, to expand the range of practitioners offering medication abortion.

## Methods

Data are reported from the Studying Accompaniment Feasibility and Effectiveness Study, a prospective observational cohort study of callers to safe abortion hotlines and accompaniment groups in Argentina, Nigeria, and a country in Southeast Asia (anonymized). Full protocol details for Argentina and Nigeria,^21^ findings from a pilot study,^19^ and main outcomes (noninferiority of self-managed abortion of pregnancy duration <9 weeks vs historical clinical controls)^18^ have been published previously. Procedures in Southeast Asia followed the same protocol as detailed in the protocol article.^21^ This study was approved by the Allendale Investigational Review Board; the Fundación Huésped institutional review board approved the Argentina-specific protocol. An independent data monitoring and oversight committee reviewed the study protocols and instruments and a planned interim analysis of safety events. Informed consent was obtained verbally. The study was prospectively registered with the ISRCTN Registry.^22^ We followed the Strengthening the Reporting of Observational Studies in Epidemiology (STROBE) reporting guidelines for observational studies and Medical Abortion Reporting of Efficacy (MARE) guideline for reporting efficacy from medication abortion studies.

### Participant Recruitment

Accompaniment groups that served as recruitment sites all provide detailed counseling on abortion options, self-assessing medication abortion eligibility, how to take abortion medications according to currently endorsed regimens, how to determine completion, and how to navigate the formal health care system if needed. During the data collection period, abortion was legally restricted at all sites and largely unavailable in the formal health care system.

Between July 31, 2019, and October 1, 2020, eligible callers were recruited into the study by accompaniment group staff at the end of initial counseling. Callers were eligible if they contacted the group for information on starting a new medication abortion process, had no contraindications to medication abortion,^19^ were aged 13 years or older, and were not currently bleeding. Eligible participants who provided verbal consent to participate completed interviewer-administered questionnaires at enrollment (baseline), approximately 1 week after taking medications (first follow-up), and approximately 3 weeks after taking medications (second follow-up). Participants used 1 of 2 World Health Organization–recommended regimens for medication abortion, depending on what was accessible in their setting: mifepristone in combination with misoprostol or misoprostol alone. This analysis is restricted to participants who reported taking misoprostol alone. Due to lack of variation in medication regimen used within each country site, we were unable to conduct a direct comparison between misoprostol-alone users and combined regimen users.

### Outcomes

The primary outcome was effectiveness, defined as abortion completion without procedural intervention, as assessed at each follow-up through the questions, “Do you think your abortion is complete?” (Argentina and Nigeria) or “Do you think you are still pregnant?” (Southeast Asia). Participants were asked how they determined that their abortion was complete and direct questions about receipt of manual vacuum aspiration or dilation and curettage or evacuation procedures. The secondary outcome was safety, which was conceptualized as an experience of potential warning signs (bleeding that soaks more than 2 pads per hour for >2 hours, pain that does not go away with pain relievers and impedes normal activities, fever >38 °C for >24 hours, and/or foul-smelling yellow or green vaginal discharge) and potential adverse events indicated by receipt of the following medical treatments: intravenous fluids, blood transfusion, and/or overnight stay in a hospital. Data collectors assessed safety outcomes using a checklist read to participants (eg, “Did you experience any of the following…?”). Other outcomes of interest included details on regimen dosing, initiation and duration of bleeding and cramping, adverse effects, time to expulsion (measured in hours from first dose of misoprostol), and health care–seeking experiences. Participants self-reported all outcomes over the phone via a standardized interviewer-administered questionnaire at first and second follow-up (eFigure 1 in Supplement 1).

### Statistical Analysis

The data analysis was performed between January 6, 2022, and September 8, 2023. We calculated proportions of outcomes, estimated 95% CIs for primary outcomes, and calculated frequencies and ranges of other outcomes (1) overall, (2) by pregnancy duration at the time of the abortion, and (3) by regimen adherence. Pregnancy duration was assessed by date of last menstrual period^23^ or ultrasound.

We compared ever seeking health care between participants who ever experienced potential warning signs and those without warning signs using χ^2^ tests of independence. Among participants who sought health care, we compared reasons for care seeking and treatment received between those who reported potential warning signs and those without warning signs using χ^2^ tests of independence. We considered a 2-sided *P* < .05 to be statistically significant.

While clinical assessment of outcomes was impossible for legal and ethical reasons, prior research has demonstrated the validity of patient self-assessment of abortion outcome.^24^ We additionally conducted a Monte Carlo sensitivity analysis^25^ to account for (1) possible misclassification of the primary outcome due to self-report and (2) selection bias from possible differential loss to follow-up (a full description of Monte Carlo sensitivity analysis methods is provided in the eMethods in Supplement 1).

Main analyses were conducted using Stata, version 15 software (StataCorp LLC). Sensitivity analyses were conducted using R, version 4.0.2 software (R Foundation for Statistical Computing).

## Results

A total of 2265 callers were screened for eligibility, of whom 1594 were eligible and 1352 consented and enrolled (85%). For this analysis, we excluded 715 callers who did not use misoprostol alone (n = 610), did not proceed with medication abortion (n = 22), or used unknown medications (n = 83) (Figure).

**Figure.**
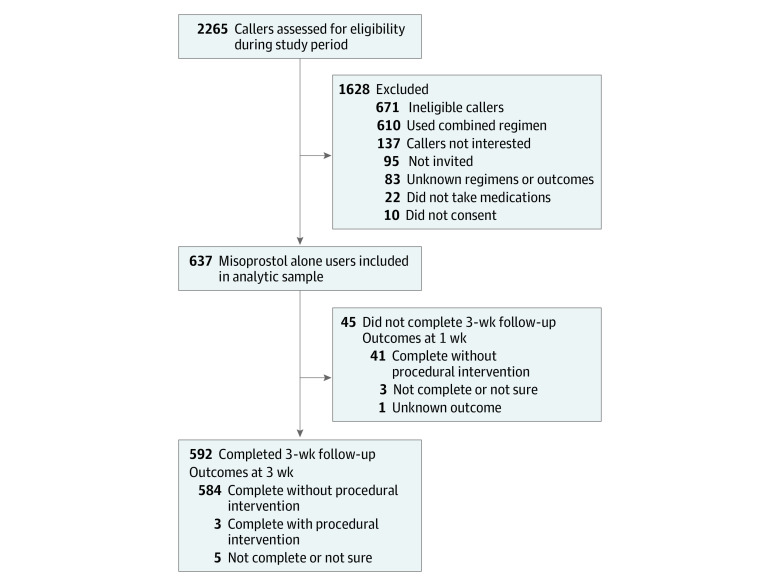
Study Profile Outcomes for callers with unknown regimens or outcomes and those who did not complete a 3-week follow-up were modeled in a Monte Carlo sensitivity analysis. Outcomes at last recorded follow-up were included from participants who completed a 3-week follow-up (n = 592) and those who only completed a 1-week follow-up (n = 45).

Sociodemographic baseline characteristics of the final analytic sample of 637 participants are provided in Table 1. A total of 591 participants were from Nigeria (92.8%), 45 (7.1%) were from Southeast Asia, and 1 (0.2%) was from Argentina. A majority of participants were aged 20 to 29 years (384 [60.2%]) and had completed secondary school or higher (616 [97.2%]). Most (617 [96.9%]) confirmed their pregnancy via a pregnancy test. Pregnancy duration was determined by date of last menstrual period (615 [96.5%]) or ultrasonography (22 [3.5%]). At the time of taking their first misoprostol dose, most participants had a pregnancy duration of less than 7 weeks (317 [49.8%]), followed by 7 to less than 9 weeks (205 [32.2%]); 115 (18.0%) had pregnancy durations 9 weeks or longer. A small proportion (35 [5.5%]) reported a prior attempt to end their pregnancy before contacting the hotline or accompaniment group.

**Table 1.** Sociodemographic Characteristics of Participants Using Misoprostol Alone for Self-Managed Medication Abortion in the Studying Accompaniment Feasibility and Effectiveness Study

Characteristic	No. (%)			*P* value^b^
	Total (N = 637)	Used currently endorsed misoprostol-alone regimen (n = 532)^a^	Used other misoprostol-alone regimen (n = 105)^a^	
Study site				
Argentina	1 (0.2)	1 (0.2)	0	<.001
Nigeria	591 (92.8)	531 (99.8)	60 (57.1)	
Southeast Asia	45 (7.1)	0	45 (42.9)	
Participant age, y				
<20	23 (3.6)	17 (3.2)	6 (5.7)	.052
20-24	157 (24.6)	123 (23.1)	34 (32.4)	
25-29	227 (35.6)	188 (35.3)	39 (37.1)	
30-34	119 (18.7)	106 (19.9)	13 (12.4)	
≥35	111 (17.4)	98 (18.4)	13 (12.4)	
Level of education				
Completed primary school	13 (2.0)	11 (2.1)	2 (1.9)	.55
Completed secondary school	263 (41.3)	226 (42.5)	37 (35.2)	
More than secondary school	356 (55.9)	295 (55.5)	61 (58.1)	
Missing	5 (0.8)	0	5 (4.8)	
Prior attempts to end current pregnancy				
No	600 (94.2)	514 (96.6)	86 (81.9)	<.001
Yes	35 (5.5)	18 (3.4)	17 (16.2)	
Missing	2 (0.3)	0	2 (1.9)	
Ascertainment of pregnancy (select all)				
Pregnancy test at home or a facility	617 (96.9)	528 (99.2)	89 (84.8)	<.001
Late or missed period	1 (0.2)	1 (0.2)	0	NA
Ultrasound	22 (3.5)	6 (1.1)	16 (14.7)	<.001
Bimanual examination	1 (0.2)	0	1 (1)	NA
Pregnancy duration at abortion, wk				
<7	317 (49.8)	281 (52.8)	36 (34.3)	<.001
7 to <9	205 (32.2)	166 (31.2)	39 (37.1)	
9 to <12	92 (14.4)	71 (13.3)	21 (20.5)	
12 to 16	23 (3.6)	14 (2.6)	9 (8.6)	

Abbreviation: NA, not applicable.

^a^ Currently endorsed misoprostol-alone regimen is 3 doses of 800 μg misoprostol, administered sublingually, vaginally, or buccally 3 hours apart. Other regimens used are reported in eTable 1 in Supplement 1.

^b^ Fisher exact test, excluding missing values.

Participants were counseled on a standard regimen, though there was variation in the actual regimen used based on medication availability and individual adherence to the regimen. Most participants (532 [83.5%]) reported taking the standard misoprostol-alone regimen of 3 doses of 800 μg misoprostol, each dose administered 3 hours apart (2400 μg misoprostol total) (eTable 1 in Supplement 1). Other regimens included additional doses of 800 μg or multiple doses of 400 μg misoprostol administered continuously until expulsion (eTable 1 in Supplement 1). Almost all participants (626 [98.3%]) took misoprostol sublingually (eTable 1 in Supplement 1). Most participants obtained misoprostol from a pharmacy (471 [73.9%]) and reported that medications were stored in a blister pack (514 [80.7%]).

At first follow-up after taking misoprostol (median, 9 days; IQR, 7-12 days), 605 of 635 participants (95.3%; 95% CI, 93.3%-96.7%) reported that their abortion was complete without procedural intervention (Table 2). At second follow-up (median, 23 days; IQR, 21-26 days), 584 of 592 participants (98.6%; 95% CI, 97.3%-99.3%) reported that their abortion was complete without procedural intervention. At last recorded follow-up (median, 22 days; IQR, 21-26 days), 625 of 637 participants (98.1%; 95% CI, 96.7%-98.9%) reported that their abortion was complete without procedural intervention. Effectiveness was higher among participants who used the currently endorsed regimen (3 doses of 800 μg misoprostol) compared with those who did not (529 participants [99.4%; 95% CI, 98.3%-99.8%] vs 96 participants [91.4%; 95% CI, 84.3%-95.5%] at last recorded follow-up) (eTables 2 and 3 in Supplement 1). Effectiveness at last follow-up was high across all pregnancy durations (<7 weeks: 315 participants [99.4%; 95% CI, 97.5%-99.8%]; 7 to <9 weeks: 200 participants [97.6%; 95% CI, 94.2%-99.0]; 9 to <12 weeks: 90 participants [97.8%; 95% CI, 91.6%-99.5%]; 12-16 weeks: 20 participants [87.0%; 95% CI, 64.9%-96.0]) (Table 2). Participants self-assessed abortion completion based on pregnancy symptoms resolving (534 [85.0%]), noticing the products of conception (415 [66.1%]), and/or negative pregnancy test results (302 [48.1%]) (eTable 4 in Supplement 1). After accounting for measurement and selection biases, the bias-adjusted effectiveness estimate remained similar to observations, conditional on chosen bias parameters (94.5%; 95% simulation interval, 91.2%-96.6%) (eTable 5 in Supplement 1).

**Table 2.** Effectiveness and Safety of Self-Managed Medication Abortion With Misoprostol Alone in the Studying Accompaniment Feasibility and Effectiveness Study

Characteristic	All participants (N = 637)		Pregnancy duration							
			<7 wk (n = 317)		7 to <9 wk (n = 205)		9 to <12 wk (n = 92)		12 to 16 wk (n = 23)	
	No.	% (95% CI)	No.	% (95% CI)	No.	% (95% CI)	No.	% (95% CI)	No.	% (95% CI)
Effectiveness at first follow-up (n = 635)^a^										
Complete without procedural intervention	605	95.3 (93.3-96.7)	305	96.2 (93.4-97.8)	194	95.1 (91.1-97.4)	85	92.4 (84.8-96.4)	21	95.5 (71.4-99.4)
Complete with procedural intervention	1	0.2 (0.0-1.1)	1	0.3 (0.0-2.2)	0	NA	0	NA	0	NA
Not complete or not sure	28	4.4 (3.1-6.3)	11	3.5 (1.9-6.2)	10	4.9 (2.6-8.9)	6	6.5 (2.9-13.9)	1	4.5 (0.6-28.6)
Missing	1	0.2 (0.0-1.1)	0	NA	0	NA	1	1.1 (0.1-7.5)	0	NA
Effectiveness at second follow-up (n = 592)^a^										
Complete without procedural intervention	584	98.6 (97.3-99.3)	296	99.3 (97.4-99.8)	185	97.9 (94.5-99.2)	84	100	19	90.5 (68.8-97.6)
Complete with procedural intervention	3	0.5 (0.2-1.6)	1	0.3 (0.0-2.4)	1	0.5 (0.1-3.7)	0	NA	1	4.8 (0.7-27.2)
Not complete or not sure	5	0.8 (0.4-2.0)	1	0.3 (0.0-2.4)	3	1.6 (0.5-4.8)	0	NA	1	4.8 (0.7-27.2)
Effectiveness at last recorded follow-up (n = 637)^a^										
Complete without procedural intervention	625	98.1 (96.7-98.9)	315	99.4 (97.5-99.8)	200	97.6 (94.2-99.0)	90	97.8 (91.6-99.5)	20	87.0 (64.9-96.0)
Complete with procedural intervention	3	0.5 (0.2-1.5)	1	0.3 (0.0-2.2)	1	0.5 (0.1-3.4)	0	NA	1	4.3 (0.5-27.5)
Not complete or not sure	8	1.3 (0.6-2.5)	1	0.3 (0.0-2.2)	4	2.0 (0.7-5.1)	1	1.1 (0.1-7.5)	2	8.7 (2.0-30.6)
Missing	1	0.2 (0.0-1.1)	0	NA	0	NA	1	1.1 (0.1-7.5)	0	NA
Potential warning signs (n = 637)^b^										
No warning signs	584	91.6 (89.4-93.7)	297	93.7 (90.4-95.9)	183	89.3 (84.2-92.8)	83	90.2 (83.5-95.6)	21	91.3 (71.1-97.8)
At least 1 potential warning sign	52	8.2 (6.3-10.6)	20	6.3 (4.1-9.6)	22	10.7 (7.2-15.8)	8	8.7 (4.4-16.7)	2	8.7 (2.0-30.6)
Bleeding more than 2 pads/h for ≥2 h	14	2.2 (1.3-3.7)	5	1.6 (0.7-3.7)	4	2.0 (0.7-5.1)	4	4.3 (1.6-11.3)	1	4.3 (0.5-27.5)
Pain that did not resolve	21	3.3 (2.2-5.0)	6	1.9 (0.9-4.2)	12	5.9 (3.3-10.1)	3	3.3 (1.1-9.9)	0	NA
Fever higher than 38 °C	4	0.6 (0.2-1.7)	2	0.6 (0.2-2.5)	0	NA	1	1.1 (0.2-7.6)	1	4.3 (0.5-27.5)
Foul discharge	23	3.6 (2.4-5.4)	10	3.2 (1.7-5.8)	9	4.4 (2.3-8.3)	4	4.3 (1.6-11.3)	0	NA
Potential adverse events (n = 637)^b^										
No potential adverse events	630	98.9 (97.7-99.5)	316	99.7 (97.8-100.0)	202	98.5 (95.5-99.5)	91	98.9 (92.7-99.8)	21	91.3 (69.4-98.0)
At least 1 potential adverse event	6	0.9 (0.4-2.1)	1	0.3 (0.0-2.2)	3	1.5 (0.5-4.5)	0	NA	2	8.7 (2.0-30.6)
Intravenous fluids	6	0.9 (0.4-2.1)	1	0.3 (0.0-2.2)	3	1.5 (0.5-4.5)	0	NA	2	8.7 (2.0-30.6)
Overnight stay	3	0.5 (0.1-1.5)	1	0.3 (0.0-2.2)	1	0.5 (0.1-3.4)	0	NA	1	4.3 (0.6-25.3)
Blood transfusion	0	NA	0	NA	0	NA	0	NA	0	NA

Abbreviation: NA, not applicable.

^a^ A total of 635 participants completed a 1-wk follow-up (first follow-up) (median, 9 days after taking medications; IQR, 7-12 days after taking medications), 592 participants completed a 3-wk follow-up (second follow-up) (median, 23 days after taking medications; IQR, 21-26 days after taking medications). Last recorded follow-up included outcome measures at 1-wk follow-up for 45 participants who did not complete a 3-wk follow-up (median time of last recorded follow-up after taking medication, 22 days; IQR, 21-26 days).

^b^ Potential warning signs and adverse events measured across both time points; data were missing for 1 participant (included in denominator).

Almost all participants (633 [99.4%]) reported experiencing at least some bleeding (Table 3). Bleeding typically started after the second dose (302 participants [47.4%]) or third dose (244 participants [38.2%]) of misoprostol, with heavy bleeding starting after the third dose for most participants (462 [72.5%]) (eFigure 2 in Supplement 1). Most participants (485 [76.2%]) reported that bleeding lasted less than 7 days (median, 4 days; IQR, 3-6 days); 557 (87.4%) reported 1 to 3 days of heavy bleeding. Almost all participants (588 [92.3%]) reported seeing the products of conception, with expulsion after the third dose (499 [78.3%]). Median time to expulsion after taking the first dose of misoprostol was 12 hours (IQR, 9-15 hours); 544 participants (85.4%) reported expelling products of conception within 24 hours of taking their first dose of misoprostol. Common side effects were nausea (335 [52.6%]), fever (232 [36.4%]), diarrhea (181 [28.4%]), and chills (161 [25.3%]). Most participants reported experiencing some type of physical pain related to their abortion process (591 [92.8%]). Most participants (575 [90.3%]) did not take anything to prevent pain prior to starting their abortion; 406 [63.7%] reported taking pain medication after their abortion started to manage their pain.

**Table 3.** Bleeding, Cramping, Expulsion, and Side Effects Among Participants Using Misoprostol Alone in the Studying Accompaniment Feasibility and Effectiveness Study

Physical experience	Pregnancy duration, No. (%)				
	All durations (N = 637)	<7 wk (n = 317)	7 to 9 wk (n = 205)	9 to <12 wk (n = 92)	12 to 16 wk (n = 23)
Duration of any bleeding, d					
0	3 (0.5)	1 (0.3)	1 (0.5)	0	1 (4.3)
1-3	182 (28.6)	96 (30.3)	53 (25.9)	25 (27.2)	8 (34.8)
4-6	300 (47.1)	155 (48.9)	93 (45.4)	44 (47.8)	8 (34.8)
7-9	89 (14.0)	45 (14.2)	30 (14.6)	11 (12.0	3 (13.0)
>10	62 (9.7)	20 (6.3)	28 (13.7)	11 (12.0)	3 (13.0)
Unknown or missing	1 (0.2)	0	0	1 (1.1)	0
Duration of heavy bleeding, d					
0	25 (3.9)	20 (6.3)	1 (0.5)	2 (2.2)	2 (8.7)
1-3	557 (87.4)	273 (86.1)	179 (87.3)	86 (93.5)	19 (82.6)
4-6	43 (6.8)	23 (7.3)	16 (7.8)	3 (3.3)	1 (4.3)
7-9	9 (1.4)	1 (0.3)	7 (3.4)	0	1 (4.3)
>10	3 (0.5)	0	2 (1.0)	1 (1.1)	0
Duration of cramping, d					
No cramping	12 (1.9)	7 (2.2)	3 (1.5)	2 (2.2)	0
<1	212 (33.3)	110 (34.7)	59 (28.8)	32 (34.8)	11 (47.8)
1-2	296 (46.5)	154 (48.6)	95 (46.3)	40 (43.5)	7 (30.4)
3	61 (9.6)	28 (8.8)	20 (9.8)	11 (12.0)	2 (8.7)
≥4	54 (8.5)	18 (5.7)	27 (13.2)	7 (7.6)	2 (8.7)
Unknown or missing	2 (0.3)	0	1 (0.5)	0	1 (4.3)
Time to expulsion, h					
Did not notice products of conception	48 (7.5)	26 (8.2)	11 (5.4)	7 (7.6)	4 (17.4)
<8	79 (12.4)	36 (11.4)	25 (12.2)	16 (17.4)	2 (8.7)
8 to ≤12	311 (48.8)	157 (49.5)	102 (49.8)	42 (45.7)	10 (43.5)
12 to ≤16	96 (15.1)	52 (16.4)	31 (15.1)	11 (12.0)	2 (8.7)
16 to ≤24	58 (9.1)	28 (8.8)	22 (10.7)	6 (6.5)	2 (8.7)
>24	44 (6.9)	18 (5.7)	14 (6.8)	9 (9.8)	3 (13.0)
Missing	1 (0.2)	0	0	1 (1.1)	0
Side effects					
Pain	591 (92.8)	301 (95.0)	189 (92.2)	81 (88.0)	20 (87.0)
Nausea	335 (52.6)	156 (49.2)	111 (54.1)	54 (58.7)	14 (60.9)
Fever	232 (36.4)	110 (34.7)	70 (34.1)	42 (45.7)	10 (43.5)
Diarrhea	181 (28.4)	78 (24.6)	69 (33.7)	28 (30.4)	6 (26.1)
Chills	161 (25.3)	71 (22.4)	58 (28.3)	27 (29.3)	5 (21.7)
Vomiting	89 (14.0)	41 (12.9)	29 (14.1)	14 (15.2)	5 (21.7)
Itchiness or hives	22 (3.5)	8 (2.5)	6 (2.9)	5 (5.4)	3 (13.0)
Difficulty breathing	1 (0.2)	0	1 (0.5)	0	0
Facial numbness	6 (0.9)	2 (0.6)	3 (1.5)	1 (1.1)	0
Sweaty hands	4 (0.6)	1 (0.3)	3 (1.5)	0	0
Did not experience any side effects	78 (12.2)	52 (16.4)	19 (9.3)	7 (7.6)	0
Missing	3 (0.5)	0	1 (0.5)	1 (1.1)	1 (4.3)

Most participants (584 [91.6%; 95% CI, 89.4%-93.7%]) did not report any warning signs that would indicate the possible need for follow-up care, such as prolonged bleeding, extreme pain, high fever, or problematic vaginal discharge (Table 2). Medical experiences that could indicate an adverse event (eg, receiving intravenous fluids, blood transfusion, or overnight hospital stay) were experienced by 6 participants (0.9%; 95% CI, 0.4%-2.1%).

Approximately one-quarter of participants (149 [23.4%]) reported seeking follow-up care; those who reported at least 1 warning sign were more likely to report seeking follow-up care than those with no warning signs (23 [44.2%] vs 126 [21.6%]; *P* < .001). Approximately one-half of participants who sought care went to a hospital or clinic (79 [53.7%]), while the rest sought care from pharmacies (Table 4). The primary reason for seeking additional follow-up care was to confirm abortion completion (133 [89.2%]). The most common medical treatment or procedure received was ultrasound examination (34 [22.8%]). Two participants (1.4%) received additional misoprostol (unknown amount). Among participants who sought care, those who reported at least 1 potential warning sign, compared with those with no warning signs, were more likely to report seeking care for concerns related to symptoms (12 [52.2%] vs 4 [3.2%]; *P* < .001) and more likely to report receiving antibiotics (9 [40.9%] vs 2 [1.6%]; *P* < .001), pain medication (9 [40.9%] vs 3 [2.4%]; *P* < .001), or intravenous fluids (4 [18.2%] vs 2 [1.6%]; *P* = .005). Participants with no warning signs were more likely to receive no medical treatment when seeking care than those with at least 1 warning sign (97 [77.0%] vs 3 [13.0%]; *P* < .001).

**Table 4.** Care-Seeking Experiences Among Participants Using Misoprostol Alone Who Sought Follow-Up Care in the Studying Accompaniment Feasibility and Effectiveness Study

Characteristic	No. (%)			*P* value^a^
	All (n = 149)	No warning signs reported (n = 126)	Reported a warning sign (n = 23)	
Type of facility				
Clinic or hospital	79 (53.0)	64 (50.8)	15 (65.2)	.40
Pharmacy or diagnostic laboratory	68 (45.6)	60 (47.6)	8 (34.8)	
Other or unknown	2 (1.3)	2 (1.6)	0	
Reason for seeking care^b^				
To confirm completion	133 (89.2)	120 (95.3)	13 (56.5)	<.001
For concerns about symptoms	16 (10.7)	4 (3.2)	12 (52.2)	<.001
Another reason	6 (4.0)	4 (3.2)	2 (8.7)	.23
Treatment received^b^				
Observation	3 (2.0)	1 (0.8)	2 (8.7)	.06
Additional misoprostol	2 (1.4)	2 (1.6)	0	>.99
Antibiotics	11 (7.4)	2 (1.6)	9 (40.9)	<.001
Pain medication	12 (8.1)	3 (2.4)	9 (40.9)	<.001
Other medications	13 (8.8)	5 (4.0)	8 (36.4)	.01
Manual vacuum aspiration	2 (1.4)	1 (0.8)	1 (4.5)	.28
Dilation and curettage	1 (0.7)	0	1 (4.5)	.15
Ultrasound	34 (22.8)	25 (19.8)	9 (40.9)	.05
Intravenous fluids	6 (4.1)	2 (1.6)	4 (18.2)	.005
Blood transfusion	0	0	0	
Overnight stay	3 (2.0)	1 (0.8)	2 (9.1)	.06
No treatment	100 (67.1)	97 (77.0)	3 (13.0)	<.001
Clinician aware of self-managed abortion				
No, I did not tell them anything	115 (79.3)	104 (84.6)	11 (50.0)	.002
No, I told them I had a miscarriage	5 (3.4)	4 (3.3)	1 (4.5)	
Yes, I told them	17 (11.7)	11 (8.9)	6 (27.3)	
Yes, they suspected or found out	4 (2.8)	2 (1.6)	2 (9.1)	
Other	4 (2.8)	2 (1.6)	2 (9.1)	

^a^ Fisher exact test.

^b^ Participants could select more than 1 response.

Almost all participants (589 [99.5%]) reported receiving all the support they needed from the hotline. When asked about preference for future abortion care, 557 participants (94.1%) reported that they would prefer to self-manage their abortion with support from the hotline, and 15 (2.5%) reported that they would prefer to go to a health facility (eTable 6 in Supplement 1).

## Discussion

This cohort study is among the few prospective studies of misoprostol-alone effectiveness using currently endorsed regimens. The findings suggest that misoprostol alone is highly effective across pregnancy durations, with low adverse events. Effectiveness of misoprostol alone may be similar to the combined regimen of mifepristone and misoprostol, especially in settings with limited access to or use of early procedural intervention.

The effectiveness reported in our study aligns with other studies of self-managed abortion with misoprostol alone (range, 88%-100%)^9,10,19,20,26^ and is higher than the effectiveness reported by comparable clinical studies (range, 84.2%-93.7%).^16,17^ This difference in observed effectiveness may be due to several study design and service delivery features. For instance, clinical studies are predisposed to early procedural intervention, may use shorter timelines to evaluate completion and intervention (1-2 weeks vs 3-4 weeks in self-management study contexts), do not always allow for additional doses of misoprostol, and may not provide comprehensive on-demand counseling and support to participants about the expected experience of misoprostol alone. As most participants in this study reported completing their abortion at 1 week using only the planned doses, it may be possible that the higher effectiveness in the context of this study is due to comprehensive counseling about what to expect and how to ascertain that the abortion is complete as well as lack of ultrasonography for assessment of completion (which may lead to potential procedural overtreatment).^27,28^

We have provided detailed data on experiences of bleeding, cramping, and side effects. These findings may provide needed evidence that can be used to update patient information forms and counseling protocols to ensure that all individuals using misoprostol alone for abortion, regardless of setting, have accurate information about what to expect. Knowing what to expect from a misoprostol-alone abortion process may help to address user expectations, calm concerns, and lead to decreased unnecessary health care seeking and overall better perceptions of quality.^29,30,31^ Reducing unnecessary health care seeking is particularly important in contexts where health care seeking after an abortion places individuals at legal risk.

### Implications

Misoprostol alone is a highly effective medication abortion regimen and warrants renewed attention. Clinicians and advocates can use these data to inform expansions of their scope of practice to include misoprostol-alone regimens for abortion where they currently do not and to advocate for decriminalization and expansion of the full spectrum of options for abortion care. Clinicians, community health care practitioners, and safe abortion hotline counselors and accompaniers alike can draw from the detailed data on physical experiences to enhance the counseling and preparedness of people initiating a misoprostol-alone abortion and thereby potentially reducing unnecessary care seeking.

These findings may contribute to growing confidence in the misoprostol-alone regimen. Even in contexts where mifepristone is available, misoprostol alone could remain an option, especially for people who want to immediately begin their abortion process (vs waiting 24-48 hours after mifepristone as required by the combined regimen). This option may be particularly salient in contexts where people need to travel for abortion care and have concerns about expelling the pregnancy en route to or at home. This study provides needed guidance on the effectiveness of this more widely available regimen, particularly in the face of increasingly stringent and severe restrictions on access to mifepristone in the US and internationally.

### Strengths and Limitations

This study has several strengths, including a large sample size, detailed and systematic data collection on previously unreported aspects of the misoprostol-alone experience (particularly physical and health care–seeking experiences), low loss to follow-up, and strong collaborative partnership^32^ in design and implementation of the study. This study also has several limitations. Due to legal restrictions on abortion access in the study sites at the time of data collection, this study used self-reported, observational outcomes rather than clinical assessment of randomly assigned study interventions. However, research has demonstrated concurrence between patient self-assessment of abortion outcome vs ultrasonography following medication abortion.^24^ Additionally, 45 participants (7.1%) did not complete a 3-week follow-up; while the majority of these participants (41 [91.1%]) reported a complete abortion without procedural abortion at 1-week follow-up, it is possible that some procedural interventions that occurred after 1-week follow-up were not captured. To account for this limitation, we conducted a Monte Carlo sensitivity analysis, which indicated that our measure of effectiveness was robust to the possible misclassification and selection factors described here.

## Conclusions

The findings of this study indicate that misoprostol-alone regimens may open opportunities for innovative access through nontraditional practitioners, pharmacists, and other contexts. As abortion access, and specifically medication abortion, comes under increasing legal attack around the world, expanding the availability of existing, evidence-based methods for medication abortion can help to ensure that all people can access abortion when and where they need it.

## Notes

Supplement 1. **eFigure 1.** Study Instrument **eMethods.** Monte Carlo Sensitivity Analysis **eTable 1.** Misoprostol-Alone Regimens Used by Participants in the SAFE Study (N = 637) **eTable 2.** Abortion Completion Among Participants Who Used Misoprostol Alone in the SAFE Study by Regimen Used (N = 637) **eTable 3.** Abortion Completion Among Nigerian Participants Who Used Standard Misoprostol-Alone Regimen in the SAFE Study by Pregnancy Duration (n = 531) **eTable 4.** Abortion Completion Among Participants Who Used Misoprostol Alone in the SAFE Study by Misoprostol Amount and Regimen (N = 637) **eTable 5.** Bias-Corrected Effectiveness From a Monte Carlo Sensitivity Analysis of Data From the SAFE Study (N = 50 000 Iterations) **eTable 6.** Support and Preferences for Future Abortion Care Among Participants Who Used Misoprostol Alone in the SAFE Study at Last Follow-Up (n = 592) **eFigure 2.** Initiation of Bleeding, Cramping, and Expulsion Among Participants Using Misoprostol Alone in the SAFE Study (N = 637)

Supplement 2.
Data Sharing Statement
