# Supplementary material for: Medication Abortion Safety and Effectiveness With Misoprostol Alone
Source: JAMA Netw Open. 2023 Oct 27;6(10):e2340042. doi: 10.1001/jamanetworkopen.2023.40042 (PMC10611991; doi:10.1001/jamanetworkopen.2023.40042)
Supplement: Supplement 1. — eFigure 1. Study Instrument eMethods. Monte Carlo Sensitivity Analysis eTable 1. Misoprostol-Alone Regimens Used by Participants in the SAFE Study (N = 637) eTable 2. Abortion Completion Among Participants Who Used Misoprostol Alone in the SAFE Study by Regimen Used (N = 637) eTable 3. Abortion Completion Among Nigerian Participants Who Used Standard Misoprostol-Alone Regimen in the SAFE Study by Pregnancy Duration (n = 531) eTable 4. Abortion Completion Among Participants Who Used Misoprostol Alone in the SAFE Study by Misoprostol Amount and Regimen (N = 637) eTable 5. Bias-Corrected Effectiveness From a Monte Carlo Sensitivity Analysis of Data From the SAFE Study (N = 50 000 Iterations) eTable 6. Support and Preferences for Future Abortion Care Among Participants Who Used Misoprostol Alone in the SAFE Study at Last Follow-Up (n = 592) eFigure 2. Initiation of Bleeding, Cramping, and Expulsion Among Participants Using Misoprostol Alone in the SAFE Study (N = 637) [file jamanetwopen-e2340042-s001.pdf]

Supplemental Online Content

Jayaweera R, Egwuatu I, Nmezi S, et al. Medication abortion safety and effectiveness with misoprostol alone. *JAMA Netw Open*. 2023;6(10):e2340042.
doi:10.1001/jamanetworkopen.2023.40042

eFigure 1. Study Instrument

eMethods. Monte Carlo Sensitivity Analysis

eTable 1. Misoprostol-Alone Regimens Used by Participants in the SAFE Study (N = 637)

eTable 2. Abortion Completion Among Participants Who Used Misoprostol Alone in the SAFE Study by Regimen Used (N = 637)

eTable 3. Abortion Completion Among Nigerian Participants Who Used Standard Misoprostol-Alone Regimen in the SAFE Study by Pregnancy Duration (n = 531)

eTable 4. Abortion Completion Among Participants Who Used Misoprostol Alone in the SAFE Study by Misoprostol Amount and Regimen (N = 637)

eTable 5. Bias-Corrected Effectiveness From a Monte Carlo Sensitivity Analysis of Data From the SAFE study (N = 50 000 Iterations)

eTable 6. Support and Preferences for Future Abortion Care Among participants Who Used Misoprostol Alone in the SAFE Study at Last Follow-Up (n = 592)

eFigure 2. Initiation of Bleeding, Cramping, and Expulsion Among Participants Using Misoprostol Alone in the SAFE Study (N = 637)

This supplemental material has been provided by the authors to give readers additional information about their work.

© 2023 Jayaweera R et al. *JAMA Network Open.*

eFigure 1. Study Instrument

SAFE 1st and 2nd Follow-Up –STUDY INSTRUMENT

1. Have you gotten the pills yet?
   - ☐ No

     If no: Why not? *Let participant answer freely, Select all that apply*
     - ☐ Decided to continue the pregnancy → END SURVEY
     - ☐ Could not find the pills
     - ☐ Did not have enough money for the pills
     - ☐ Not enough time to go get the pills
     - ☐ Had a miscarriage so no longer need the pills → END SURVEY
     - ☐ Concerns about using the pills
     - ☐ I am not sure I am still pregnant
     - ☐ Other ____
     - ☐ No response

     For everyone who did not report deciding to continue the pregnancy or miscarriage: Despite these challenges, do you still plan to get the pills and take them?
     - ☐ Yes
     - ☐ No
     - ☐ Don't know
     - ☐ No response

       If **yes**, when do you PLAN to take the pills? ____/____/________
       dd mm yyyy

       If **no** or **don't know**, please tell me a bit more about why you do not, or are not sure if you plan to get the pills and take them [open response]:

     IF PARTICIPANT DID NOT GET THE PILLS AND DOES NOT PLAN TO TAKE THE PILLS, END SURVEY HERE. IF THEY PLAN TO GET THE PILLS, SCHEDULE NEXT FOLLOW-UP FOR 7 DAYS AFTER THEY PLAN GET/TAKE PILLS.
   - ☐ Yes

     If yes: When did you get them? ____/____/________
     dd mm yyyy

     If yes: Where did you get them? *Select all that apply*
     - ☐ Pharmacy

       If pharmacy: How many pharmacies did you need to go to before you got all of the pills that you needed? _____________
     - ☐ A friend
     - ☐ Ordered from the internet
     - ☐ Health care provider
     - ☐ Other ____
     - ☐ No response

     If yes: How were they stored?
     - ☐ Loose pills
     - ☐ Blister pack
     - ☐ Other _________________________
     - ☐ No response

     If yes: What was the brand name? *If more than one brand was purchased, select all that apply.*
     - ☐ Cytotec
     - ☐ Mariprist
     - ☐ Mife Kit
     - ☐ Mife Pack

© 2023 Jayaweera R et al. *JAMA Network Open.*

- ☐ Misoclear
- ☐ Misofem
- ☐ Mistol
- ☐ Other: ____________________
- ☐ Don't know
- ☐ No response

If yes: How did you pay for the pills? (the abortion pills only) ______________________

2. Have you taken the pills yet?

☐ No

If no: Why not? *Let participant answer freely, Select all that apply*.

- ☐ Decided to continue the pregnancy → END SURVEY
- ☐ Had a miscarriage so no longer need the pills → END SURVEY
- ☐ Concerns about using the pills
- ☐ Haven't had time yet
- ☐ I am not sure I am still pregnant
- ☐ Other (specify) ____
- ☐ No response

If no: Do you plan to take the pills?

- ☐ Yes

  If yes: When do you plan to take the pills? ____/____/________
  dd mm yyyy

- ☐ No
- ☐ No response

IF PARTICIPANT HAS NOT TAKEN THE PILLS, END SURVEY HERE.

☐ Yes

If yes: What regimen did you use?

- ☐ Misoprostol alone
- ☐ Mifepristone + Misoprostol
- ☐ No response

3. In total, how many medication doses did the woman report taking? ___________________________

Dose Timing

4. **1st dose:** Which medication did you take? ☐ Mife ☐ Miso ☐ No response

How many pills did you take? __________ pills

Route of administration:

- ☐ Oral
- ☐ Buccal
- ☐ Sublingual
- ☐ Vaginal
- ☐ Other ____
- ☐ No response

What date did you take this dose?: ____/____/________ What time did you take this dose?__________
dd mm yyyy *time*

5. **2nd dose:** Which medication did you take? ☐ Mife ☐ Miso ☐ No response

How many pills did you take? __________ pills

Route of administration:

- ☐ Oral
- ☐ Buccal
- ☐ Sublingual

© 2023 Jayaweera R et al. *JAMA Network Open.*

☐ Vaginal
☐ Other ____
☐ No response

What date did you take this dose?: ____/____/________ *dd mm yyyy* What time did you take this dose?_________ *time*

6. **3rd dose:** Which medication did you take? ☐ Mife ☐ Miso ☐ No response
   How many pills did you take? _________ pills
   Route of administration:
   ☐ Oral
   ☐ Buccal
   ☐ Sublingual
   ☐ Vaginal
   ☐ Other ____
   ☐ No response
   What date did you take this dose?: ____/____/________ *dd mm yyyy* What time did you take this dose?_________ *time*
7. **4th dose:** Which medication did you take? ☐ Mife ☐ Miso ☐ No response
   How many pills did you take? _________ pills
   Route of administration:
   ☐ Oral
   ☐ Buccal
   ☐ Sublingual
   ☐ Vaginal
   ☐ Other ____
   ☐ No response
   What date did you take this dose?: ____/____/________ *dd mm yyyy* What time did you take this dose?_________ *time*
8. **5th dose:** Which medication did you take? ☐ Mife ☐ Miso ☐ No response
   How many pills did you take? _________ pills
   Route of administration:
   ☐ Oral
   ☐ Buccal
   ☐ Sublingual
   ☐ Vaginal
   ☐ Other ____
   ☐ No response
   What date did you take this dose?: ____/____/________ *dd mm yyyy* What time did you take this dose?_________ *time*
9. Did you (or do you) need to take any additional doses?
   ☐ Yes
   ☐ No
   ☐ No response

   If yes: Give details of the medicine, number of pills, route, date, and timing.

   ____

   ____

   ____

   ____

   ____

Bleeding

© 2023 Jayaweera R et al. *JAMA Network Open.*

10. Did you experience any bleeding?
    - ☐ No
    - ☐ No response
    - ☐ Yes

    If yes*:* When did you first notice ANY bleeding?
    - ☐ After the first dose of the medication
    - ☐ After the second dose of the medication
    - ☐ After the third dose of medication
    - ☐ After the fourth dose of medication
    - ☐ After the fifth dose of medication
    - ☐ After the sixth dose of medication
    - ☐ After the seventh dose of medication
    - ☐ After the eighth dose of medication
    - ☐ I don't remember
    - ☐ No response

    If yes: Over how many DAYS did you have ANY bleeding? (days) ______________________

    If yes: Was the bleeding continuous throughout this period, or did it stop and start multiple times?
    - ☐ Continuous
    - ☐ Stop and start
    - ☐ No response

    If yes*:* Over how many DAYS did you have THICK/HEAVY bleeding? (days) _______________

    If greater than 0: When did you first notice the THICK/HEAVY bleeding?
    - ☐ After the first dose of the medication
    - ☐ After the second dose of the medication
    - ☐ After the third dose of medication
    - ☐ After the fourth dose of medication
    - ☐ After the fifth dose of medication
    - ☐ After the sixth dose of medication
    - ☐ After the seventh dose of medication
    - ☐ After the eighth dose of medication
    - ☐ I don't remember
    - ☐ No response

<u>Cramping</u>

11. Did you experience any cramping?
    - ☐ No
    - ☐ No response
    - ☐ Yes

    If yes: When did you first notice ANY cramping/contractions?
    - ☐ After the first dose of the medication
    - ☐ After the second dose of the medication
    - ☐ After the third dose of medication
    - ☐ After the fourth dose of medication
    - ☐ After the fifth dose of medication
    - ☐ After the sixth dose of medication
    - ☐ After the seventh dose of medication
    - ☐ After the eighth dose of medication
    - ☐ I don't remember
    - ☐ No response

    If yes: Over how many hours did you have cramping/contractions? (hours) _____________

    If yes: Over how many days did you have cramping/contractions? (days) _______________

    If yes: Was the cramping continuous throughout this period, or did it stop and start multiple times?

© 2023 Jayaweera R et al. *JAMA Network Open.*

- ☐ Continuous
- ☐ Stop and start
- ☐ No response

Pain

12. Did you do anything to prevent pain BEFORE you started the process? (Like taking pills, watching a movie, using a heating pad, shower or bath, massage, special teas, etc.)
    - ☐ No
    - ☐ No response
    - ☐ Yes

    If yes: What did you do? *Let participant answer freely, Select all that apply.*
        - ☐ Took painkillers
        - ☐ Watched a movie/tv
        - ☐ Took a shower
        - ☐ Used a heating pad
        - ☐ Used massage
        - ☐ Took herbs
        - ☐ Drank tea
        - ☐ Listened to music
        - ☐ Other ____________________
        - ☐ No response

13. Did you feel any physical pain during the process?
    - ☐ No
    - ☐ No response
    - ☐ Yes

    If yes: Did you do anything to alleviate the pain, once it began? (Like taking pills, watching a movie/tv, using a heating pad, shower or bath, massage, special teas, etc.)
        - ☐ No
        - ☐ No response
        - ☐ Yes

        If yes: What did you do? *Select all that apply.*
            - ☐ Took painkillers
            - ☐ Watched a movie/tv
            - ☐ Took a shower
            - ☐ Used a heating pad
            - ☐ Used massage
            - ☐ Took herbs
            - ☐ Drank tea
            - ☐ Listened to music
            - ☐ Other ____________________
            - ☐ No response

Side Effects and Completion

14. During or after your process, did you experience any of the following? *Read all options, select all that apply.*
    - ☐ Nausea
    - ☐ Diarrhea
    - ☐ Vomiting
    - ☐ Fever
    - ☐ Chills

© 2023 Jayaweera R et al. *JAMA Network Open.*

- ☐ Itchiness/hives
- ☐ Difficulty breathing
- ☐ Face numbness
- ☐ Client didn't experience any of these symptoms
- ☐ No response

15. During or after the process, did you experience: *Read all options, Select all that apply*
    - ☐ Bleeding that soaked more than 2 pads per hour for more than 2 hours
    - ☐ Pain that didn't go away with pain relievers and made it difficult to do normal activities
    - ☐ Fever higher than 38C for more than 24 hours
    - ☐ Foul smelling yellow/green discharge
    - ☐ Client didn't experience any symptoms
    - ☐ No response

16. Do you feel that your abortion process is complete?
- ☐ No response
- ☐ Unsure

  If you are unsure: Why are you not sure?

- ☐ No

  If no: Why do you feel that your abortion process is not complete? *Let participant answer freely, then Select all that apply.*
  - ☐ Counselor told me I was STILL pregnant
  - ☐ Pregnancy symptoms did NOT go away
  - ☐ Doctor/nurse told me I was STILL pregnant
  - ☐ I did NOT feel the pregnancy come out
  - ☐ I did NOT see the gestational sac
  - ☐ POSITIVE pregnancy test at facility, blood
  - ☐ POSITIVE pregnancy test at facility, urine
  - ☐ POSITIVE pregnancy test, home
  - ☐ Ultrasound
  - ☐ Other ______________________________
  - ☐ No response
- ☐ Yes

  If yes: Why do you feel that your abortion is complete? *Let participant answer freely, then Select all that apply..*
  - ☐ Counselor told me I was no longer pregnant
  - ☐ Pregnancy symptoms went away
  - ☐ Doctor/nurse told me I was no longer pregnant
  - ☐ I felt the pregnancy come out
  - ☐ I saw the gestational sac
  - ☐ NEGATIVE pregnancy test at facility, blood
  - ☐ NEGATIVE pregnancy test at facility, urine
  - ☐ NEGATIVE pregnancy test, home
  - ☐ Ultrasound
  - ☐ Other ______________________________
  - ☐ No response

17. Since taking the medications, have you had an ultrasound?
    - ☐ Yes

      If yes*:* What was the result of the ultrasound?

© 2023 Jayaweera R et al. *JAMA Network Open.*

- ☐ Complete abortion
- ☐ Incomplete abortion
- ☐ Ongoing pregnancy
- ☐ Other ____
- ☐ No response

If yes: What was the date of the ultrasound? ____/____/________
dd mm yyyy

18. At any point in the process, did you notice (feel or see) the pregnancy come out/expel?
    - ☐ No
    - ☐ Don't know
    - ☐ No Response
    - ☐ Yes

    If yes: At what point in the process did you notice the pregnancy (products of conception) come out?
    - ☐ After the first dose of medication
    - ☐ After the second dose of medication
    - ☐ After the third dose of medication
    - ☐ After the fourth dose of medication
    - ☐ After the fifth dose of medication
    - ☐ After the sixth dose of medication
    - ☐ After the seventh dose of medication
    - ☐ After the eighth dose of medication
    - ☐ I don't remember
    - ☐ No response

    If yes: Approximately how many HOURS after your first dose of medication did the pregnancy come out? (hours)_____________

19. At any point during or after your abortion process, did you seek care at a health facility?
    - ☐ No→ Go to Q20 (Emotions)
    - ☐ No response → Go to Q20 (Emotions)
    - ☐ Yes

    If yes: Why did you seek care at a health facility? *Select all that apply.*
    - ☐ To confirm abortion completion
    - ☐ Concern about bleeding
    - ☐ Concern about pain
    - ☐ Concern about fever
    - ☐ Concern about discharge
    - ☐ Concern about nausea
    - ☐ Concern about diarrhea
    - ☐ For MVA
    - ☐ For D&C
    - ☐ Other ____
    - ☐ No response

    If yes: Did the clinicians keep you under observation? (i.e. asked you to stay for some time so they could continue to assess your symptoms, without actively treating you)
    - ☐ Yes
    - ☐ No
    - ☐ I don't know
    - ☐ No response

    If yes to seeking care at a health facility: Did they give you misoprostol?
    - ☐ Yes

© 2023 Jayaweera R et al. *JAMA Network Open.*

- ☐ No
- ☐ I don't know
- ☐ No response

If yes: Did they give you antibiotics?
- ☐ Yes
- ☐ No
- ☐ I don't know
- ☐ No response

If yes: Did they give you pain medications?
- ☐ Yes
- ☐ No
- ☐ I don't know
- ☐ No response

If yes: Did they give you other medications (beyond miso, antibiotics, or pain medications)?
- ☐ Yes (Specify which medications) ________________________________
- ☐ No
- ☐ I don't know
- ☐ No response

If yes: Did you have an MVA?
- ☐ Yes
- ☐ No
- ☐ I don't know
- ☐ No response

If yes: Did you have a D&C?
- ☐ Yes
- ☐ No
- ☐ I don't know
- ☐ No response

If yes: Did they do an ultrasound at the health facility?
- ☐ Yes
- ☐ No
- ☐ I don't know
- ☐ No response

If yes: Did they give you IV fluids?
- ☐ Yes
- ☐ No
- ☐ I don't know
- ☐ No response

If yes: Did you receive a blood transfusion?
- ☐ Yes
- ☐ No
- ☐ I don't know
- ☐ No response

If yes: Did you stay overnight at the health facility?
- ☐ Yes
- ☐ No
- ☐ No response

If yes: Did you receive any other type of treatment that we haven't listed?
- ☐ Yes (specify what kind of medical treatment)________________________
- ☐ No
- ☐ I don't know
- ☐ No response

© 2023 Jayaweera R et al. *JAMA Network Open.*

If yes to seeking care at a health facility: What type of facility did you go to?

- ☐ Government/public clinic
- ☐ Private clinic
- ☐ Government/public hospital
- ☐ Private hospital
- ☐ Other ______________________________
- ☐ No response

If yes: Did the doctor or nurse know you had taken anything to try to end your pregnancy?

- ☐ Yes, I told them

  If yes, I told them: Why did you tell the provider? *Let participant answer freely, Select all that apply.*

  - ☐ They asked me directly if I had done anything
  - ☐ I wanted them to have all of the information
  - ☐ I felt comfortable sharing the information
  - ☐ I knew the provider
  - ☐ I trusted the provider
  - ☐ I felt that I had to tell the provider
  - ☐ Other ______________________________
  - ☐ No response
- ☐ Yes, they suspected/found out
- ☐ No, I told them I had a miscarriage → see below
- ☐ No, I didn't tell them anything → see below

  If "No, I told them I had a miscarriage" and "No, I didn't tell them anything" : Why did you not tell the provider? *Let participant answer freely, Select all that apply.*

  - ☐ I was afraid
  - ☐ I knew the provider personally
  - ☐ There was no medical need to tell them
  - ☐ I did not want to be judged
  - ☐ Other ______________________________
  - ☐ No response
- ☐ Other______________________________
- ☐ No response

20. What are the top 3 emotions you feel now about your abortion experience? To be clear, we mean the top 3 emotions you feel about the abortion (not about having an unwanted pregnancy).
    - ☐ Relief
    - ☐ Guilty
    - ☐ Calm
    - ☐ Happy
    - ☐ Satisfied
    - ☐ Anxious
    - ☐ Nervous
    - ☐ Relaxed
    - ☐ Fear
    - ☐ Sadness
    - ☐ Disappointment
    - ☐ Anguish
    - ☐ No emotion
    - ☐ Other ______________________________
    - ☐ No response

END OF SURVEY

© 2023 Jayaweera R et al. *JAMA Network Open.*

eMethods. Monte Carlo Sensitivity Analysis

We conducted a Monte Carlo sensitivity analysis based on data from the Studying Accompaniment model Feasibility and Effectiveness Study (the SAFE Study), to generate bias-adjusted estimates of effectiveness of self-managed medication abortion using misoprostol alone regimens.

Bias framework

Selection bias could arise if those lost to follow up were more or less likely to have a complete abortion than those who remain in the study. Misclassification of the outcome (a three level variable: complete without surgical intervention, complete with surgical intervention, not complete/unsure) may arise because measurement relies on self-report. Participants may unintentionally misclassify themselves as "complete," when their abortion process is not actually complete, or, may misclassify themselves as "not complete" when their abortion process is truly complete. We do not think participants would intentionally misrepresent their outcome. The extent of misclassification depends on sensitivity (*Se*) and specificity (*Sp*) of self-report as a method of abortion completion assessment. We do not know *Se* or *Sp* for self-reporting a complete abortion without surgical intervention. However, we assume any abortion process that ended in surgical intervention was reported with 100% accuracy as participants were directly asked if they had any surgical procedure, and intentional misclassification is unlikely. Consequently, we assume the outcome "complete with surgical intervention" is measured with 100% Sensitivity and Specificity.

Bias-adjustment methods

To calculate bias-adjusted measures of effectiveness from our measure of observed effectiveness based on the above bias framework, we adjusted for each source of bias in the reverse order it occurred. We adapted the approach described in "Accounting for Misclassification and Selection Bias in Estimating Effectiveness of Self-managed Medication Abortion," *Epidemiology* (2023); equations and assumptions briefly summarized below.

In the below equations, $\mathbf{Y} = (\mathrm{Y}_1, \mathrm{Y}_2, \mathrm{Y}_3)$ is a multinomially distributed random variable indexed by category i, where $i = 1,2,3$ and $n = \sum_{i=1}^{3} y_i$, where y_i denotes the realized outcome in category i. Category 1 (y_1) represents individuals who had a complete abortion without surgical intervention, y_2 represents those did not have a complete abortion or were unsure, and y_3 represents those who had a complete abortion with surgical intervention. The probability of each outcome is given by π_i, with $\sum_{i=1}^{3} \pi_i = 1$.

The parameter π_1 represents the probability of a complete abortion without surgical intervention (effectiveness), and is estimated by $\hat{\pi}_1 = y_1/n$. Instead of the true counts $\{y_i\}_{(i=1,2,3)}$ we observed reported counts $\{\tilde{y}_i\}_{(i=1,2,3)}$, which we adjusted for misclassification and selection bias below.

Adjustment for misclassification

For those who do not report surgical intervention ($i = 1,2$), we adjusted for misclassification of self-reported abortion completion without surgical intervention using assumed Sensitivity (*Se)* and Specificity (*Sp). Se* refers to the proportion of those with a complete abortion who self-reported their abortion was complete; *Sp* refers to the proportion of those without a complete abortion who

© 2023 Jayaweera R et al. *JAMA Network Open.*

reported their abortion was not complete or were unsure. Given assumed *Se* and *Sp* of self-reported outcomes among those who did not have a surgical intervention, we calculated outcomes y_i' that are adjusted for eligibility and misclassification (see Lash et al, Ch. 6, Equation 6.12 for derivation):[20]

$$y_1' = \frac{\tilde{y}_1 - (\tilde{y}_1 + \tilde{y}_2) \times (1 - Sp)}{Se - (1 - Sp)} \tag{Eq. 1}$$

We made no adjustment to the number who report a complete abortion with surgical intervention ($y_3' = \tilde{y}_3$), and adjusted the number who report their abortion is not complete or not sure ($y_2' = \tilde{n} - y_1' - y_3'$). This serves to preserve the total number of participants so $n' = \tilde{n}$.

<u>Adjustment for selection bias</u>

To adjust for selection bias from differential loss to follow-up and differential enrollment, we calculated probabilities of inclusion in the final analytic sample (denoted *S*) given outcome $i = k$, as the joint probability of completing follow-up and enrolling in the study:

$$\Pr[S = 1 | i = k] = \Pr[F = 1 | E = 1, i = k] \times \Pr[E = 1 | i = k] \tag{Eq. 2}$$

where *F* and *E* are indicators of completing follow-up and study enrollment, respectively.

Using Bayes Rule, and assuming counts $\{y_i'\}_{i=1}^{3}$ adjusted for eligibility and misclassification, we calculated the probability of follow-up given enrollment and outcome type $i = k$:

$$\Pr[F = 1 \mid E = 1, i = k] = \frac{y_k'}{y_k' + (n - n')\Pr[i = k | F = 0, E = 1]} \tag{Eq. 3}$$

where *n* is the total number of participants enrolled, and $(n - n')\Pr[y_k' | F = 0, E = 1]$ is the expected number of participants lost to follow-up in outcome group *k*. The relevant bias parameters in this step are:

$$\theta_{k|F,E} = \Pr[i = k | F = 0, E = 1] \tag{Eq. 4}$$

$$\theta_{E|k} = \Pr[E = 1 | i = k] \tag{Eq. 5}$$

© 2023 Jayaweera R et al. *JAMA Network Open.*

for the probability of outcome *k* among those lost to follow-up (Eq. 4) and the probability of enrollment (Eq. 5). Substituting these parameters into Equations 2 and 3, the probability of inclusion $\Pr[S = 1|i = k]$, denoted by $\theta_{S|k}$, can be expressed as:

$$\theta_{S|k} = \frac{y'_k}{y'_k + (n - n')\theta_{k|F,E}} \times \theta_{E|k} \qquad \text{(Eq. 6)}$$

<u>Estimation of bias-adjusted measures of effectiveness</u>

Based on the above, we calculated bias-adjusted measures of effectiveness using the equations below.

Observed effectiveness is:

$$\tilde{\pi}_1 = \frac{\tilde{y}_1}{n} \qquad \text{(Eq. 7)}$$

Effectiveness adjusted for eligibility and misclassification is:

$$\pi'_1 = \frac{y'_1}{n'} \qquad \text{(Eq. 8)}$$

Our estimate of effectiveness, adjusted for misclassification and selection bias by inversely weighting participants based on probability of inclusion ($\omega_k = 1/\theta_{S,k}$), is the proportion:

$$\pi_1^{\dagger} = \frac{y'_1 \times \omega_1}{(y'_1 \times \omega_1) + (y'_2 \times \omega_2) + (y'_3 \times \omega_3)} \qquad \text{(Eq. 9)}$$

<u>Selection of bias parameters</u>

To provide effectiveness estimates that are adjusted for possible sources of bias, estimates of the following bias parameters are needed: sensitivity of self-report of abortion completion (*Se*), specificity of self-report of abortion completion (*Sp*), proportion of participants lost to follow-up who had a complete abortion without surgical intervention ($\theta_{1,F,E} = \Pr[y'_1|F = 0, E = 1]$, probability of enrolling in the study ($\theta_{E,k} = \Pr[E = 1|i = k]$), and probability of being ineligible for the study among those reporting a complete abortion ($\theta_{N,1} = \Pr[\text{not pregnant} \mid i = 1]$). As the true value of the above parameters are unknown, we assume the above parameters are drawn from Beta distributions, defined by shape parameters alpha and beta (α, β), and described below. Shape parameters for each distribution are listed in Table 1; median and interquartile ranges of the underlying distributions are described in eAppendix1.

Se and Sp of self-report: Estimates of *Se* and *Sp* for self-reported abortion outcomes were drawn from a validation study assessing women's self assessment of medication abortion success via a symptom checklist (gold standard: provider determination of success). *Se* from the study was 92% (95% CI 90, 93); *Sp* from the study was 44% (95% CI: 36, 51). For this MCSA, we drew values of *Se* from a Beta[90,10] distribution left-truncated at $\tilde{\pi}_1$ (the observed effectiveness) and *Sp* from a Beta[10,90] distribution left-truncated at $1 - \tilde{\pi}_1$ using the *rtrunc* function from the *truncdist* package in R.[21] We used truncated distributions for these parameters to avoid negative cell counts in the bias correction.

© 2023 Jayaweera R et al. *JAMA Network Open.*

We chose parameters corresponding to a lower specificity to be more conservative in our assumptions.

Probability of inclusion: Probabilities of enrollment based on abortion outcome were drawn from a Beta [85,15] distribution; we assumed no differences in enrollment based on eventual abortion outcome. After the study period, the study team was able to reach 47 out of the 90 individuals who did not complete any follow-up. We also conservatively assumed those missing a second follow-up to be lost to follow-up (45 individuals). We conservatively assumed effectiveness among those lost to follow-up of 70%, and drew values of effectiveness among those lost to follow-up from a Beta [70, 30] distribution.

Monte Carlo sampling

We calculated adjusted measures of effectiveness (π_1', and $\pi_1^\dagger$) based on equations 8 and 9, across 50,000 simulations with bias parameters drawn from the above specified Beta distributions (see below). The distribution of these bias-adjusted point estimates only characterize uncertainty given the distributions of the bias parameters; to additionally account for random error, we resampled the log-odds of effectiveness from a normal distribution with mean equal to the point estimate of the bias-adjusted log odds, and standard deviation equal to its standard error.[14,22] We present 95% simulation-based confidence intervals based on 2.5% and 97.5% quantiles; point estimates represent the median estimate across simulations (below). All analyses were completed in R 4.0.2;[23] code is available at (https://github.com/ruvaniJ/SAFE).

Distribution of bias parameters across 50,000 simulations

	**Parameter distributions**	**Simulated distributions**
**Bias Parameters**	**Beta(α, β) [min, max]**	**Median % (95% Interval)**
Sensitivity of self-reported abortion completion	Beta(90,10) [OE[a], 1]	98.8 (98.7, 99.2)
Specificity of self-reported abortion completion	Beta(10,90)[1-OE[a],1]	9.7 (5.0, 16.5)
Probability of complete abortion among those lost to follow-up	Beta(70,30)[0,1]	70.0 (60.1, 78.5)
Probability of enrolling (complete without surgical)	Beta(85,15)[0,1]	85.2 (77.3, 91.3)
Probability of enrolling (complete with surgical)	Beta(85,15)[0,1]	85.2 (77.4, 91.3)
Probability of enrolling (not complete)	Beta(85,15)[0,1]	85.2 (77.5, 91.3)
Overall probability of inclusion (complete without surgical)	See Equation 6	92.1 (90.5, 93.7)
Overall probability of inclusion (complete with surgical)	See Equation 6	10.7 (8.9, 13.2)
Overall probability of inclusion (not complete)	See Equation 6	13.7 (0.6, 47.0)

a Sensitivity and specificity are mathematically minimally bounded by observed effectiveness (98.1%) and 1 - observed effectiveness (1.9%), respectively.

eTable 1. Misoprostol Alone Regimens Used by Participants in the SAFE Study (N = 637)

	All pregnancy durations (n = 637)		Pregnancy Duration at start of abortion process							
			< 7 weeks (n = 317)		[7-9) weeks (n = 205)		[9-12) weeks (n = 92)		[12-16] weeks (n = 23)	
**Characteristics**	**n**	**%**	**n**	**%**	**n**	**%**	**n**	**%**	**n**	**%**
**Where pills were obtained from**										
Pharmacy	471	73.9	257	81.1	141	68.8	58	63	15	65.2
A friend	83	13	35	11	27	13.2	19	20.7	2	8.7
Ordered from the internet	6	0.9	0	0	4	2	1	1.1	1	4.3
Health care provider	19	3	8	2.5	6	2.9	5	5.4	0	0
Other	72	11.3	26	8.2	31	15.1	11	12	4	17.3
Other	58	9.1	26	8.2	23	11.2	8	8.7	1	4.3
Mama Miso	14	2.2	0	0	8	3.9	3	3.3	3	13
**How pills were packaged**										
Loose pills	78	12.2	38	12	21	10.2	17	18.5	2	8.7
Blister pack	514	80.7	273	86.1	161	78.5	64	69.6	16	69.6
Unknown	46	7.3	7	2.2	23	11.2	11	12	5	21.7
**Regimen (condensed)**										
800ug	3	0.5	0	0	3	1.5	0	0	0	0
800ug + 800 ug (2 doses)	12	1.9	6	1.9	3	1.5	3	3.3	0	0
800 ug + 800ug + 800ug (3 doses)	532	83.5	281	88.6	166	81	71	77.2	14	60.9
Other*	90	14.1	30	9.5	33	16.1	18	19.6	9	39.1
**Amount of misoprostol taken for first dose**										
Less than 800 mcg	35	5.5	19	6	9	4.4	4	4.3	3	13
800 mcg	602	94.5	298	94	196	95.6	88	95.7	20	87
**Total amount of misoprostol taken**										
Less than 800 mcg	1	0.2	1	0.3	0	0	0	0	0	0
800 mcg	7	1.1	3	0.9	4	2	0	0	0	0
1000 - 1400 mcg	26	4.1	11	3.5	7	3.4	6	6.5	2	8.7
1600 - 2200 mcg	51	8	14	4.4	18	8.8	14	15.2	5	21.7
2400 mcg	542	85.1	284	89.6	172	83.9	71	77.2	15	65.2
> 2400 mcg	10	1.6	4	1.3	4	2	1	1.1	1	4.3
**Regimen**										
800 ug + 800ug + 800ug (recommended)	532	83.5	281	88.6	166	81	71	77.2	14	60.9
800 ug + 400ug + 400ug + 400ug	13	2	1	0.3	6	2.9	4	4.3	2	8.7
800ug + 800 ug	12	1.9	6	1.9	3	1.5	3	3.3	0	0
800 ug + 200ug	11	1.7	3	0.9	5	2.4	3	3.3	0	0
400ug + 400ug + 400ug	8	1.3	3	0.9	2	1	1	1.1	2	8.7
800ug + 400ug + 400ug	8	1.3	0	0	4	2	2	2.2	2	8.7
600 ug + 800ug + 800ug	7	1.1	3	0.9	3	1.5	0	0	1	4.3
800 ug + 400ug + 400ug + 400ug + 400ug	5	0.8	1	0.3	3	1.5	0	0	1	4.3
800 ug + 800ug + 800ug + 800ug	5	0.8	2	0.6	1	0.5	1	1.1	1	4.3
600 ug + 600ug + 600ug	4	0.6	2	0.6	1	0.5	1	1.1	0	0
600 ug + 600ug	3	0.5	2	0.6	0	0	1	1.1	0	0
800 ug	3	0.5	0	0	3	1.5	0	0	0	0
800ug + 400 ug + 800ug + 400ug	3	0.5	1	0.3	2	1	0	0	0	0
200ug + 200ug + 200ug + 200ug	2	0.3	1	0.3	1	0.5	0	0	0	0
800 ug + 400ug + 800ug	2	0.3	1	0.3	0	0	1	1.1	0	0

© 2023 Jayaweera R et al. *JAMA Network Open.*

Characteristics	All pregnancy durations (n = 637)		Pregnancy Duration at start of abortion process							
			< 7 weeks (n = 317)		[7-9) weeks (n = 205)		[9-12) weeks (n = 92)		[12-16] weeks (n = 23)	
	n	%	n	%	n	%	n	%	n	%
200ug x 12 doses	1	0.2	1	0.3	0	0	0	0	0	0
200ug + 200ug + 200ug	1	0.2	1	0.3	0	0	0	0	0	0
200ug + 200ug + 800ug	1	0.2	0	0	0	0	1	1.1	0	0
200ug + 400ug + 400ug	1	0.2	1	0.3	0	0	0	0	0	0
200ug + 400ug + 800ug	1	0.2	1	0.3	0	0	0	0	0	0
400ug + 400ug	1	0.2	1	0.3	0	0	0	0	0	0
400ug + 400ug + 400ug + 400ug	1	0.2	1	0.3	0	0	0	0	0	0
400ug + 400ug + 400ug + 400ug + 400ug	1	0.2	0	0	1	0.5	0	0	0	0
600ug + 200ug	1	0.2	1	0.3	0	0	0	0	0	0
600ug + 400ug	1	0.2	1	0.3	0	0	0	0	0	0
600ug x 6 doses	1	0.2	0	0	1	0.5	0	0	0	0
800ug + 600ug	1	0.2	0	0	0	0	1	1.1	0	0
800ug + 600ug + 600ug	1	0.2	0	0	0	0	1	1.1	0	0
800ug + 800ug + 400ug	1	0.2	0	0	0	0	1	1.1	0	0
800ug + 800ug + 400ug + 400ug	1	0.2	0	0	1	0.5	0	0	0	0
800ug + 800ug + 600ug + 800ug + 800ug + 800ug	1	0.2	1	0.3	0	0	0	0	0	0
800ug + 800ug + 800ug + 800ug + 800ug	1	0.2	0	0	1	0.5	0	0	0	0
800ug + 800ug + 800ug + 800ug + 800ug + 800ug	1	0.2	1	0.3	0	0	0	0	0	0
800ug + 800ug + 1000ug	1	0.2	0	0	1	0.5	0	0	0	0
**Route of administration (1st dose)**										
Oral	7	1.1	2	0.6	4	2	1	1.1	0	0
Sublingual	626	98.3	313	98.7	200	97.6	90	97.8	23	100
Vaginal	3	0.5	1	0.3	1	0.5	1	1.1	0	0
Other	1	0.2	1	0.3	0	0	0	0	0	0
**Route of administration (2nd dose)***										
Oral	7	1.1	2	0.6	4	2.0	1	1.1	0	0
Sublingual	624	98.4	313	98.7	197	97.5	91	98.9	23	100
Vaginal	2	0.3	1	0.3	1	0.5	0	0.0	0	0
Other	1	0.2	1	0.3	0	0.0	0	0.0	0	0
**Route of administration (3rd dose)***										
Oral	5	0.8	1	0.3	3	1.5	1	1.2	0	0
Sublingual	599	99.2	302	99.7	191	98.5	83	98.8	23	100

*3 participants who did not take a second dose & 33 participants who did not take a third dose excluded

© 2023 Jayaweera R et al. *JAMA Network Open.*

eTable 2. Abortion Completion Among Participants Who Used Misoprostol Alone in the SAFE Study by Regimen (N = 637)

	All participants (n = 637)		Misoprostol alone regimen			
			Used endorsed regimen (3 doses 800ug) (n = 532)		Used other misoprostol alone regimen (n = 105)	
**Characteristic**	**n**	**%, (95% CI)**	**n**	**%, (95% CI)**	**n**	**%, (95% CI)**
**Effectiveness at one week follow-up (n = 635)**						
Complete without procedural intervention	605	95.3 (93.3, 96.7)	511	96.1 (94, 97.4)	94	91.3 (84, 95.4)
Complete with procedural intervention	1	0.2 (0, 1.1)	1	0.2 (0, 1.3)	0	0 (0, 0)
Not complete/Not sure	28	4.4 (3.1, 6.3)	20	3.8 (2.4, 5.8)	8	7.8 (3.9, 14.8)
Missing	1	0.2 (0, 1.1)	0	0 (0, 0)	1	1 (0.1, 6.6)
**Effectiveness at three week follow-up (n = 592)**						
Complete without procedural intervention	584	98.6 (97.3, 99.3)	494	99.8 (98.6, 100)	90	92.8 (85.6, 96.5)
Complete with procedural intervention	3	0.5 (0.2, 1.6)	1	0.2 (0, 1.4)	2	2.1 (0.5, 7.9)
Not complete/Not sure	5	0.8 (0.4, 2)	0	0 (0, 0)	5	5.2 (2.2, 11.8)
**Effectiveness at last recorded follow-up (n=637)**						
Complete without procedural intervention	625	98.1 (96.7, 98.9)	529	99.4 (98.3, 99.8)	96	91.4 (84.3, 95.5)
Complete with procedural intervention	3	0.5 (0.2, 1.5)	1	0.2 (0, 1.3)	2	1.9 (0.5, 7.3)
Not complete/Not sure	8	1.3 (0.6, 2.5)	2	0.4 (0.1, 1.5)	6	5.7 (2.6, 12.2)
Missing	1	0.2 (0, 1.1)	0	-	1	1 (0.1, 6.5)
**Potential Warning Signs* (n = 637)**						
No warning signs	584	91.6 (89.4, 93.7)	502	94.4 (92, 96)	83	79.1 (69.8, 85.7)
At least one potential warning sign	52	8.16 (6.3, 10.6)	30	5.6 (4, 8)	22	20.9 (14.3, 30.2)
Bleeding more than 2 pads/hr for 2+ hrs	14	2.2 (1.3, 3.7)	5	0.9 (0.4, 2.2)	9	8.7 (4.5, 15.9)
Pain that did not resolve	21	3.3 (2.2, 5)	12	2.3 (1.3, 3.9)	9	8.7 (4.5, 15.9)
Fever higher than 38C	4	0.6 (0.2, 1.7)	3	0.6 (0.2, 1.7)	1	1 (0.1, 6.6)
Foul discharge	23	3.6 (2.4, 5.4)	16	3 (1.8, 4.9)	7	6.7 (3.2, 13.6)
**Potential Adverse Events* (n = 637)**						
No potential adverse events	630	98.9 (97.7, 99.5)	531	99.8 (98.7, 100)	100	95.2 (89, 98)
At least one potential adverse event	6	0.9 (0.4, 2.1)	1	0.2 (0, 1.3)	5	4.8 (2, 11)
IV fluids	6	0.9 (1.8, 8.8)	1	0.2 (0, 1.3)	5	4.8 (2, 11)
Overnight stay	3	0.5 (0.6, 6.1)	1	0.2 (0, 1.3)	2	1.9 (0.5, 7.4)
Blood tranfusion	0	-	0	-	0	-

*reported at either time point

© 2023 Jayaweera R et al. *JAMA Network Open.*

eTable 3. Abortion Completion Among Nigerian Participants Who Used Standard Misoprostol-Alone Regimen in the SAFE Study by Pregnancy Duration (n = 531)

			Pregnancy Duration (N = 531)							
	All gestations (n = 531)		< 7 weeks (n = 281)		[7-9) weeks (n = 166)		[9-12) weeks (n = 70)		[12-14] weeks (n = 14)	
**Characteristic**	**n**	**%, (95% CI)**	**n**	**%, (95% CI)**	**n**	**%, (95% CI)**	**n**	**%, (95% CI)**	**n**	**%, (95% CI)**
**Effectiveness at one week follow-up (n = 531)**										
Complete without surgical intervention	510	96 (94, 97.4)	271	96.4 (93.5, 98.1)	158	95.2 (90.6, 97.6)	67	95.7 (87.5, 98.6)	14	100 (0, 0)
Complete with surgical intervention	1	0.2 (0, 1.3)	1	0.4 (0, 2.5)	0	-	0	-	0	-
Not complete/Not sure	20	3.8 (2.4, 5.8)	9	3.2 (1.7, 6)	8	4.8 (2.4, 9.4)	3	4.3 (1.4, 12.5)	0	-
**Effectiveness at three week follow-up (n = 494)**										
Complete without surgical intervention	493	99.8 (98.6, 100)	263	99.6 (97.3, 99.9)	152	100	64	100	14	100
Complete with surgical intervention	1	0.2 (0, 1.4)	1	0.4 (0.1, 2.7)	0	-	0	-	0	-
Not complete/Not sure	0	-	0	-	0	-	0	-	0	-
**Potential Warning Signs***										
No warning signs	502	94.5 (92.2, 96.2)	266	94.7 (91.3, 96.8)	155	93.4 (88.4, 96.3)	68	97.1 (89.3, 99.3)	13	92.9 (62.9, 99)
At least one potential warning sign	29	5.5 (3.8, 7.8)	15	5.3 (3.2, 8.7)	11	6.6 (3.7, 11.6)	2	2.9 (0.7, 10.7)	1	7.1 (1, 37.1)
Bleeding more than 2 pads/hr for 2+ hrs	5	0.9 (0.4, 2.2)	3	1.1 (0.3, 3.3)	1	0.6 (0.1, 4.2)	1	1.4 (0.2, 9.5)	0	0 (0, 0)
Pain that did not resolve	12	2.3 (1.3, 3.9)	6	2.1 (1, 4.7)	5	3 (1.3, 7)	1	1.4 (0.2, 9.5)	0	0 (0, 0)
Fever higher than 38C	3	0.6 (0.2, 1.7)	1	0.4 (0, 2.5)	0	0 (0, 0)	1	1.4 (0.2, 9.5)	1	7.1 (1, 37.1)
Foul discharge	15	2.8 (1.7, 4.6)	8	2.8 (1.4, 5.6)	6	3.6 (1.6, 7.8)	1	1.4 (0.2, 9.5)	0	0 (0, 0)
**Potential Adverse Events***										
No potential adverse events	530	99.8 (98.7, 100)	280	99.6 (97.5, 100)	166	100	70	100	14	100
At least one potential adverse event	1	0.2 (0, 1.3)	1	0.4 (0, 2.5)	0	-	0	-	0	-
IV fluids	1	0.2 (0, 1.3)	1	0.4 (0, 2.5)	0	-	0	-	0	-
Overnight stay	1	0.2 (0, 1.3)	1	0.4 (0, 2.5)	0	-	0	-	0	-
Blood transfusion	0	-	0	-	0	-	0	-	0	-

*reported at either time point

© 2023 Jayaweera R et al. *JAMA Network Open.*

eTable 4. Abortion Completion Among Participants Who Used Misoprostol Alone in the SAFE Study by Misoprostol Amount and Regimen (N = 637)

	All participants (n = 637)		Pregnancy Duration							
			< 7 weeks (n = 317)		[7-9) weeks (n = 205)		[9-12) weeks (n = 92)		[12-16] weeks (n = 23)	
**Abortion completion**	**n**	**%**	**n**	**%**	**n**	**%**	**n**	**%**	**n**	**%**
**At first follow-up (n = 635)**										
Complete without surgical intervention	605	95.3	305	96.2	194	95.1	85	92.4	21	95.5
Complete with surgical intervention	1	0.2	1	0.3	0	0	0	0	0	0
Not complete/Not sure	28	4.4	11	3.5	10	4.9	6	6.5	1	4.5
Missing	1	0.2	0	0.0	0	0.0	1	1.1	0	0.0
**How did you know your abortion was complete (n = 606)***										
Counselor told me I was no longer pregnant	9	1.5	2	0.7	7	3.6	0	0	0	0
Pregnancy symptoms went away	520	85.8	266	86.9	161	83	75	88.2	18	85.7
Doctor/nurse told me I was no longer pregnant	6	1	1	0.3	1	0.5	2	2.4	2	9.5
I felt the pregnancy come out	407	67.2	212	69.3	127	65.5	55	64.7	13	61.9
I saw the gestational sac	24	4	3	1	12	6.2	7	8.2	2	9.5
NEGATIVE pregnancy test at facility, blood	87	14.4	49	16	23	11.9	12	14.1	3	14.3
NEGATIVE pregnancy test at facility, urine	26	4.3	12	3.9	10	5.2	3	3.5	1	4.8
NEGATIVE pregnancy test, home	196	32.3	97	31.7	60	30.9	31	36.5	8	38.1
Negative pregnancy test (any)	288	47.5	147	48	88	45.4	42	49.4	11	52.4
Ultrasound	30	5	12	3.9	9	4.6	6	7.1	3	14.3
Other	60	9.9	31	10.1	24	12.4	3	3.5	2	9.5
**At last follow-up (n = 637)**										
Complete without surgical intervention	625	98.1	315	99.4	200	97.6	90	97.8	20	87
Complete with surgical intervention	3	0.5	1	0.3	1	0.5	0	0	1	4.3
Not complete/Not sure	8	1.3	1	0.3	4	2	1	1.1	2	8.7
Missing	1	0.2	0	0.0	0	0.0	1	1.1	0	0.0
**How did you know your abortion was complete (n = 628)***										
Counselor told me I was no longer pregnant	10	1.6	2	0.6	8	4	0	0	0	0
Pregnancy symptoms went away	534	85	271	85.8	167	83.1	78	86.7	18	85.7
Doctor/nurse told me I was no longer pregnant	6	1	1	0.3	1	0.5	2	2.2	2	9.5
I felt the pregnancy come out	415	66.1	214	67.7	130	64.7	58	64.4	13	61.9
I saw the gestational sac	24	3.8	4	1.3	11	5.5	7	7.8	2	9.5
NEGATIVE pregnancy test at facility, blood	93	14.8	52	16.5	25	12.4	13	14.4	3	14.3
NEGATIVE pregnancy test at facility, urine	26	4.1	12	3.8	10	5	3	3.3	1	4.8
NEGATIVE pregnancy test, home	204	32.5	100	31.6	63	31.3	33	36.7	8	38.1
Negative pregnancy test (any)	302	48.1	153	48.4	93	46.3	45	50	11	52.4
Ultrasound	31	4.9	13	4.1	9	4.5	6	6.7	3	14.3
Other	62	9.9	33	10.4	24	11.9	3	3.3	2	9.5

© 2023 Jayaweera R et al. *JAMA Network Open.*

(continued)

Abortion completion	Regimen							
	Other (n = 90)		1 800 ug dose (n = 3)		2 800 ug doses (n = 12)		3 800 ug doses (n = 532)	
	n	%	n	%	n	%	n	%
**At first follow-up (n = 635)**								
Complete without surgical intervention	80	90.9	2	66.7	12	100	511	96.1
Complete with surgical intervention	0	0	0	0	0	0	1	0.2
Not complete/Not sure	7	8	1	33.3	0	0	20	3.8
Missing	1	1.1	0.0	0.0	0.0	0.0	0	0.0
**How did you know your abortion was complete (n = 606)***								
Counselor told me I was no longer pregnant	7	8.8	0.0	0.0	0.0	0.0	2	0.4
Pregnancy symptoms went away	62	77.5	1.0	50.0	8.0	66.7	449	87.7
Doctor/nurse told me I was no longer pregnant	4	5.0	0.0	0.0	0.0	0.0	2	0.4
I felt the pregnancy come out	26	32.5	0.0	0.0	5.0	41.7	376	73.4
I saw the gestational sac	23	28.7	0.0	0.0	0.0	0.0	1	0.2
NEGATIVE pregnancy test at facility, blood	9	11.3	0.0	0.0	1.0	8.3	77	15.0
NEGATIVE pregnancy test at facility, urine	4	5.0	0.0	0.0	0.0	0.0	22	4.3
NEGATIVE pregnancy test, home	29	36.3	1.0	50.0	9.0	75.0	157	30.7
Negative pregnancy test (any)	40	50.0	1.0	50.0	10.0	83.3	237	46.3
Ultrasound	11	13.8	0.0	0.0	0.0	0.0	19	3.7
Other	13	16.3	1.0	50.0	1.0	8.3	45	8.8
**At last follow-up (n = 637)**								
Complete without surgical intervention	81	90	3	100	12	100	529	99.4
Complete with surgical intervention	2	2.2	0	0	0	0	1	0.2
Not complete/Not sure	6	6.7	0	0	0	0	2	0.4
Missing	1	1.1	0.0	0.0	0.0	0.0	0	0.0
**How did you know your abortion was complete (n = 628)***								
Counselor told me I was no longer pregnant	7	8.4	1.0	33.3	0.0	0.0	2	0.4
Pregnancy symptoms went away	62	74.7	2.0	66.7	8.0	66.7	462	87.2
Doctor/nurse told me I was no longer pregnant	4	4.8	0.0	0.0	0.0	0.0	2	0.4
I felt the pregnancy come out	26	31.3	1.0	33.3	5.0	41.7	383	72.3
I saw the gestational sac	23	27.7	0.0	0.0	0.0	0.0	1	0.2
NEGATIVE pregnancy test at facility, blood	9	10.8	0.0	0.0	1.0	8.3	83	15.7
NEGATIVE pregnancy test at facility, urine	4	4.8	0.0	0.0	0.0	0.0	22	4.2
NEGATIVE pregnancy test, home	33	39.8	1.0	33.3	9.0	75.0	161	30.4
Negative pregnancy test (any)	44	53.0	1.0	33.3	10.0	83.3	247	46.6
Ultrasound	11	13.3	0.0	0.0	0.0	0.0	20	3.8
Other	14	16.9	1.0	33.3	1.0	8.3	46	8.7

© 2023 Jayaweera R et al. *JAMA Network Open.*

(continued)

Abortion completion	Less than 800 mcg (n = 1)		800 mcg (n = 7)		1000 - 1400 mcg (n = 27)		1600 - 2200 mcg (n = 48)		2400 mcg (n = 542)		> 2400 mcg (n = 10)	
	n	%	n	%	n	%	n	%	n	%	n	%
**At first follow-up (n = 635)**												
Complete without surgical intervention	1	100	6	85.7	26	96.3	43	89.6	519	95.8	10	100
Complete with surgical intervention	0	0	0	0	0	0	0	0	1	0.2	0	0
Not complete/Not sure	0	0	1	14.3	1	3.7	4	8.3	22	4.1	0	0
Missing	0	0	0	0	0	0	1	2.1	0	0	0	0
**How did you know your abortion was complete (n = 606)***												
Counselor told me I was no longer pregnant	0	0	0	0	3	12	3	6.8	3	0.6	0	0
Pregnancy symptoms went away	0	0	3	50	20	80	32	72.7	456	87.7	9	90
Doctor/nurse told me I was no longer pregnant	0	0	0	0	1	4	2	4.5	3	0.6	0	0
I felt the pregnancy come out	0	0	2	33.3	5	20	16	36.4	377	72.5	7	70
I saw the gestational sac	0	0	0	0	7	28	14	31.8	3	0.6	0	0
NEGATIVE pregnancy test at facility, blood	1	100	1	16.7	5	20	3	6.8	77	14.8	0	0
NEGATIVE pregnancy test at facility, urine	0	0	1	16.7	1	4	0	0	23	4.4	1	10
NEGATIVE pregnancy test, home	0	0	2	33.3	10	40	21	47.7	160	30.8	3	30
Negative pregnancy test (any)	1	100	3	50	15	60	24	54.5	241	46.3	4	40
Ultrasound	1	100	2	33.3	3	12	5	11.4	19	3.7	0	0
Other	1	100	2	33.3	6	24	4	9.1	45	8.7	2	20
**At last follow-up (n = 637)**												
Complete without surgical intervention	1	100	7	100	25	96.2	46	90.2	536	98.9	10	100
Complete with surgical intervention	0	0	0	0	1	3.8	1	2	1	0.2	0	0
Not complete/Not sure	0	0	0	0	0	0	3	5.9	5	0.9	0	0
Missing	0	0	0	0	0	0	1	2	0	0	0	0
**How did you know your abortion was complete (n = 628)***												
Counselor told me I was no longer pregnant	0	0	1	14.3	3	11.5	3	6.4	3	0.6	0	0
Pregnancy symptoms went away	0	0	4	57.1	20	76.9	33	70.2	468	87.2	9	90
Doctor/nurse told me I was no longer pregnant	0	0	0	0	1	3.8	2	4.3	3	0.6	0	0
I felt the pregnancy come out	0	0	3	42.9	5	19.2	17	36.2	383	71.3	7	70
I saw the gestational sac	0	0	0	0	8	30.8	13	27.7	3	0.6	0	0
NEGATIVE pregnancy test at facility, blood	1	100	1	14.3	5	19.2	3	6.4	83	15.5	0	0
NEGATIVE pregnancy test at facility, urine	0	0	1	14.3	1	3.8	0	0	23	4.3	1	10
NEGATIVE pregnancy test, home	0	0	2	28.6	11	42.3	24	51.1	164	30.5	3	30
Negative pregnancy test (any)	1	100	3	42.9	16	61.5	27	57.4	251	46.7	4	40
Ultrasound	1	100	2	28.6	3	11.5	5	10.6	20	3.7	0	0

Amount of Misoprostol (spanning header over all dose columns)

© 2023 Jayaweera R et al. *JAMA Network Open.*

Other	1	100	2	28.6	6	23.1	4	8.5	47	8.8	2	20

*Participants could select multiple options

© 2023 Jayaweera R et al. *JAMA Network Open.*

eTable 5. Bias-Corrected Effectiveness From a Monte Carlo Sensitivity Analysis of Data From the SAFE Study (N = 50 000 Iterations)

	Effectiveness	
**Estimate**	**%**	**95% Intervals[a]**
Observed	98.1	96.7, 98.9
Adjustment for misclassification	98.7	95.5, 99.7
Adjusted for misclassification + selection	94.5	91.2, 96.6

[a] Observed intervals are 95% confidence intervals, adjusted intervals are 95% simulation intervals based on 2.5% and 97.5% quantiles across 50,000 simulations

© 2023 Jayaweera R et al. *JAMA Network Open.*

eTable 6. Support and Preferences for Future Abortion Care Among Participants Who Used Misoprostol Alone in the SAFE Study at Last Follow-Up (n = 592)

	All participants who completed second follow-up (n = 592)		Abortion outcome					
			Complete without surgical intervention (n = 584)		Complete with surgical intervention (n = 3)		Not complete/Not sure (n = 5)	
**Characteristic**	**n**	**%**	**n**	**%**	**n**	**%**	**n**	**%**
**Received needed support from hotline**								
No	2	0.3	1	0.2	1	33.3	0	0
Yes	589	99.5	582	99.7	2	66.7	5	100
Missing	1	0.2	1	0.2	0	0	0	0
**Received needed support from others**								
No	43	7.3	42	7.2	0	0	1	20
Yes	547	92.4	540	92.5	3	100	4	80
Missing	2	0.3	2	0.3	0	0	0	0
**Preferred future abortion care**								
In a health facility	15	2.5	11	1.9	2	66.7	2	40
Self-managed abortion with support from hotline	557	94.1	554	94.9	1	33.3	2	40
Self-managed abortion on my own	8	1.4	7	1.2	0	0	1	20
Other	9	1.5	9	1.5	0	0	0	0
Missing	0	0	0	0	0	0	0	0

© 2023 Jayaweera R et al. *JAMA Network Open.*

eFigure 2. Initiation of Bleeding, Cramping, and Expulsion Among Participants Using Misoprostol Alone in the SAFE Study (N = 637)

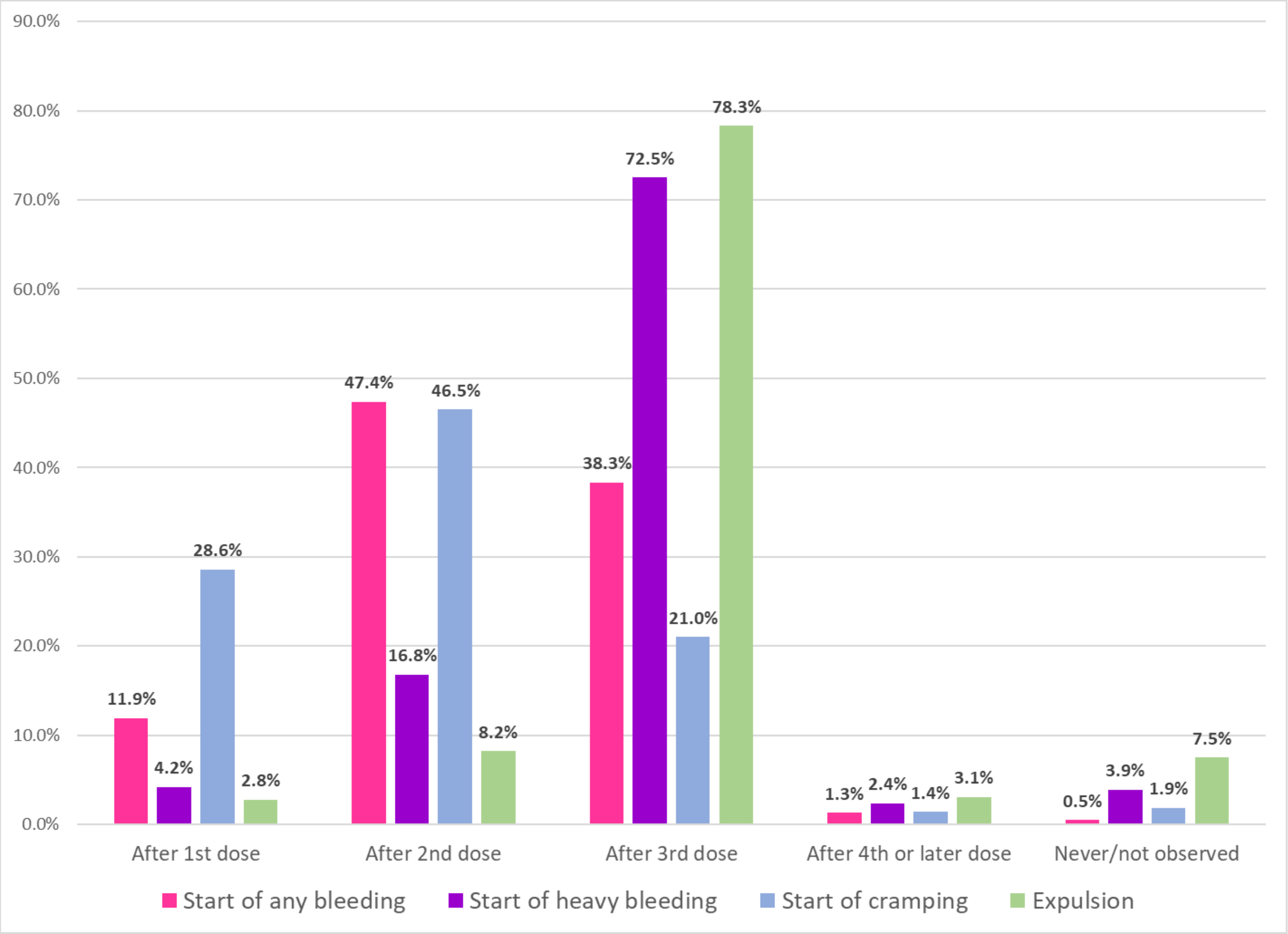

© 2023 Jayaweera R et al. *JAMA Network Open.*
