# Supplementary material for: Medication Abortion Safety and Effectiveness With Misoprostol Alone
Source: JAMA Netw Open. 2023 Oct 27;6(10):e2340042. doi: 10.1001/jamanetworkopen.2023.40042 (PMC10611991; doi:10.1001/jamanetworkopen.2023.40042)
Supplement: Supplement 2. — Data Sharing Statement [file jamanetwopen-e2340042-s002.pdf]

Data Sharing Statement

Jayaweera. Medication Abortion Safety and Effectiveness With Misoprostol Alone. *JAMA Netw Open*. Published October 27, 2023. doi:10.1001/jamanetworkopen.2023.40042

Data
Data available: No

Additional Information
Explanation for why data not available: Data is available upon reasonable request; all data requests will be reviewed by the study's Data Monitoring and Oversight Committee.
